# Analysing published global Ebola Virus Disease research using social network analysis

**DOI:** 10.1371/journal.pntd.0005747

**Published:** 2017-10-09

**Authors:** Christiane Hagel, Felix Weidemann, Stephan Gauch, Suzanne Edwards, Peter Tinnemann

**Affiliations:** 1 Institute for Social Medicine, Epidemiology and Health Economics, Charité-Universitätsmedizin Berlin, Berlin, Germany; 2 Department for Infectious Diseases Epidemiology, Robert Koch-Institut, Berlin, Germany; 3 Department for Research System and Science Dynamics, German Centre for Higher Education Research and Science Studies, Berlin, Germany; 4 Department of Health Care Management, Berlin University of Technology, Berlin, Germany; Armed Forces Health Surveillance Center, UNITED STATES

## Abstract

**Introduction:**

The 2014/2015 West African Ebola Virus Disease (EVD) outbreak attracted global attention. Numerous opinions claimed that the global response was impaired, in part because, the EVD research was neglected, although quantitative or qualitative studies did not exist. Our objective was to analyse how the EVD research landscape evolved by exploring the existing research network and its communities before and during the outbreak in West Africa.

**Methods/ Principal findings:**

Social network analysis (SNA) was used to analyse collaborations between institutions named by co-authors as affiliations in publications on EVD. Bibliometric data of publications on EVD between 1976 and 2015 was collected from Thomson Reuters’ Web of Science Core Collection (WoS). Freely available software was used for network analysis at a global-level and for 10-year periods. The networks are presented as undirected-weighted graphs. Rankings by degree and betweenness were calculated to identify central and powerful network positions; modularity function was used to identify research communities. Overall 4,587 publications were identified, of which 2,528 were original research articles. Those yielded 1,644 authors’ affiliated institutions and 9,907 connections for co-authorship network construction. The majority of institutions were from the USA, Canada and Europe. Collaborations with research partners on the African continent did exist, but less frequently. Around six highly connected organisations in the network were identified with powerful and broker positions. Network characteristics varied widely among the 10-year periods and evolved from 30 to 1,489 institutions and 60 to 9,176 connections respectively. Most influential actors are from public or governmental institutions whereas private sector actors, in particular the pharmaceutical industry, are largely absent.

**Conclusion/ Significance:**

Research output on EVD has increased over time and surged during the 2014/2015 outbreak. The overall EVD research network is organised around a few key actors, signalling a concentration of expertise but leaving room for increased cooperation with other institutions especially from affected countries. Finding innovative ways to maintain support for these pivotal actors while steering the global EVD research network towards an agenda driven by agreed, prioritized needs and finding ways to better integrate currently peripheral and newer expertise may accelerate the translation of research into the development of necessary live saving products for EVD ahead of the next outbreak.

## Author summary

Ebola Virus Disease (EVD) research publications were used to analyse and visualise collaborations between institutions jointly publishing research results, using freely available social network analysis tools. Constructed co-authorship networks between author affiliated institutions showed EVD research publications increased and networks evolved over time. The global network is organised around a few co-authoring, mostly publicly financed key actors, highly connected with powerful and broker positions. The results present an extensive narrative how modern empirical scientific methods for data processing and translation can supplement evidence-based arguments for public discussion on the status and focus of global EVD research. Based on the network characteristics or concentration of expertise, we recommend a globally agreed and prioritized EVD research agenda may facilitate the translation of this research into new EVD tools. Also, to analyse research networks regularly to enable public discussion on the direction in which research could be organized and optimised. We would like to encourage others to utilize our methods with open access tools to enhance new methods to the field of NTD R&D.

## Introduction

The 2014/2015 West African Ebola Virus Disease (EVD) outbreak with more than 28,000 cases and 11,000 deaths, was a public health emergency of international concern [1,2]. Although EVD was discovered in the former Zaire (now: Democratic Republic of Congo) more than 40 years ago, the absence of treatment generated global alarm and raised questions on the state of EVD research. Studies analysing EVD transmission and clinical trials testing EVD treatments or vaccines have been difficult due to the small number of infected cases in previous outbreaks [3,4]. Moreover, the pharmaceutical industry has been criticized for neglecting EVD research because it is not profitable enough as EVD occurred rarely and mostly in impoverished African communities [3,5–7]. EVD outbreaks have attracted general public attention since the mid-90s, benefitting science funding, leading to increased publications, but EVD research funding is mostly spent outside of affected African countries and research capacity building there was neglected [8].

The World Health Organization (WHO) called for greater transparency and better sharing of results from clinical trials as being a necessary contribution to facilitate research and development (R&D) for the benefit of science and patients [9] and published a research priority agenda [10]. The necessity for increased transparency also applies to any existing EVD research and expertise to improve the value and efficiency of research efforts.

In order to enhance the understanding of on-going EVD research activities and its communities, social network analysis (SNA) of bibliometric data of EVD related scientific publications can be used. Since co-authorships are the most visible and accessible indicator for collaborations, co-authorship-based SNA studies can be used to measure the presence of research collaborations and their evolution over time [11–13]. SNA metrics can reveal network patterns and identify its most central and influential actors [14–16].

The volume of publications, in combination with results from a co-authorship network analysis, can serve as a proxy indicator for R&D. Besides mapping the research landscape [17], especially co-authorship network analysis can provide insight into the degree of research governance and be relevant for strategic research planning [18,19]. Moreover, information from collaboration networks can be used to identify potential collaborations in order to improve research communication and therefore maybe also influence research outcomes [12,20].

The aim of this study is to identify EVD research activities and to analyse the structure of the evolving EVD research community network over time to map existing research collaborations and influential actors based on centrality network metrics.

## Methods

Based on bibliometric data we analysed the development of EVD research in two steps. Firstly, we measured the annual EVD research publications amongst all published materials. Secondly, we conducted a co-authorship network analysis at institutional level based on original research publications between 1976 and 2015. Additionally, network analyses were conducted for 10-years’ time periods in order to assess temporal network dynamics.

### Data

#### EVD research publications

Bibliometric data was collected on 17 January 2016 from Thomson Reuters’ Web of Science Core Collection (WoS) using the research query “Ebola*”. Earlier piloted information retrieval strategies included different terms, synonyms or abbreviations (e.g., EBV, EBOV or SUDV) did not reveal additional search results.

For the analysis of research publications we included publications of all document types available in the data source published between 1976 and up to 2015. For the network analysis data was restricted to original research articles from non-anonymous authors only (i.e. by excluding reviews, letters, editorial material, news items, meeting abstracts, notes, corrections, reprints, biographical items and book reviews).

**Fig 1:**
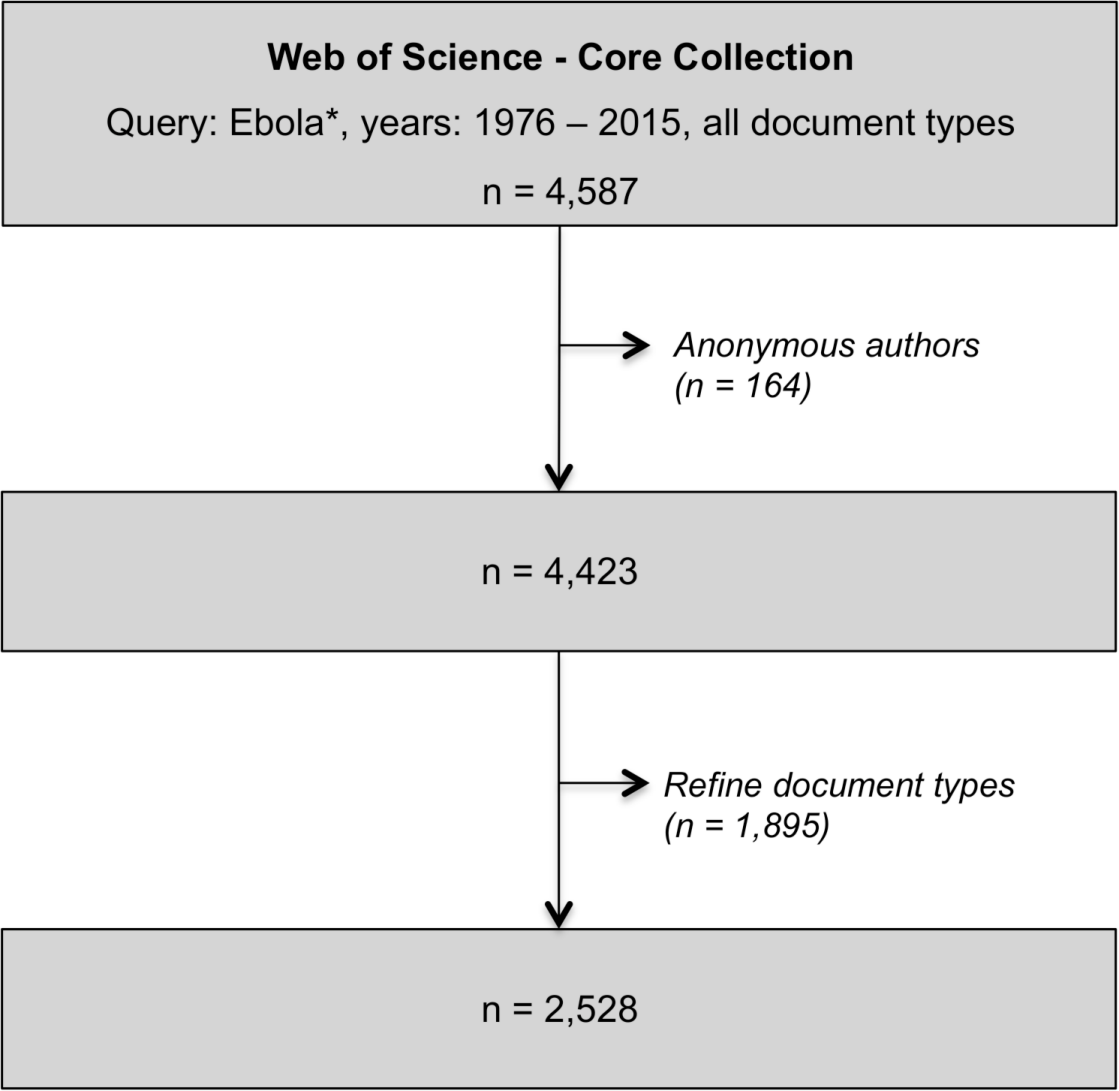
Flow chart, Web of Science Core Collection search results filtered stepwise (by years, author and document type).

In order to achieve a data demarcation, the initial data set was stepwise filtered by years from 1976 until 2015. Anonymous authors were deleted and document types restricted to original research articles (Fig 1).

#### EVD cases

For relating the EVD publications to occurrences of EVD outbreaks we collected WHO data on reported EVD cases from the online statistics portal Statista [21]. EVD case data for 2001 and 2002 (124 cases) was only available in aggregated form, therefore data for both years was divided equally (62 cases/year). For 2014 and 2015 EVD case figures were extracted from the WHO situation reports and manually calculated [1].

### Data processing

**Fig 2:**
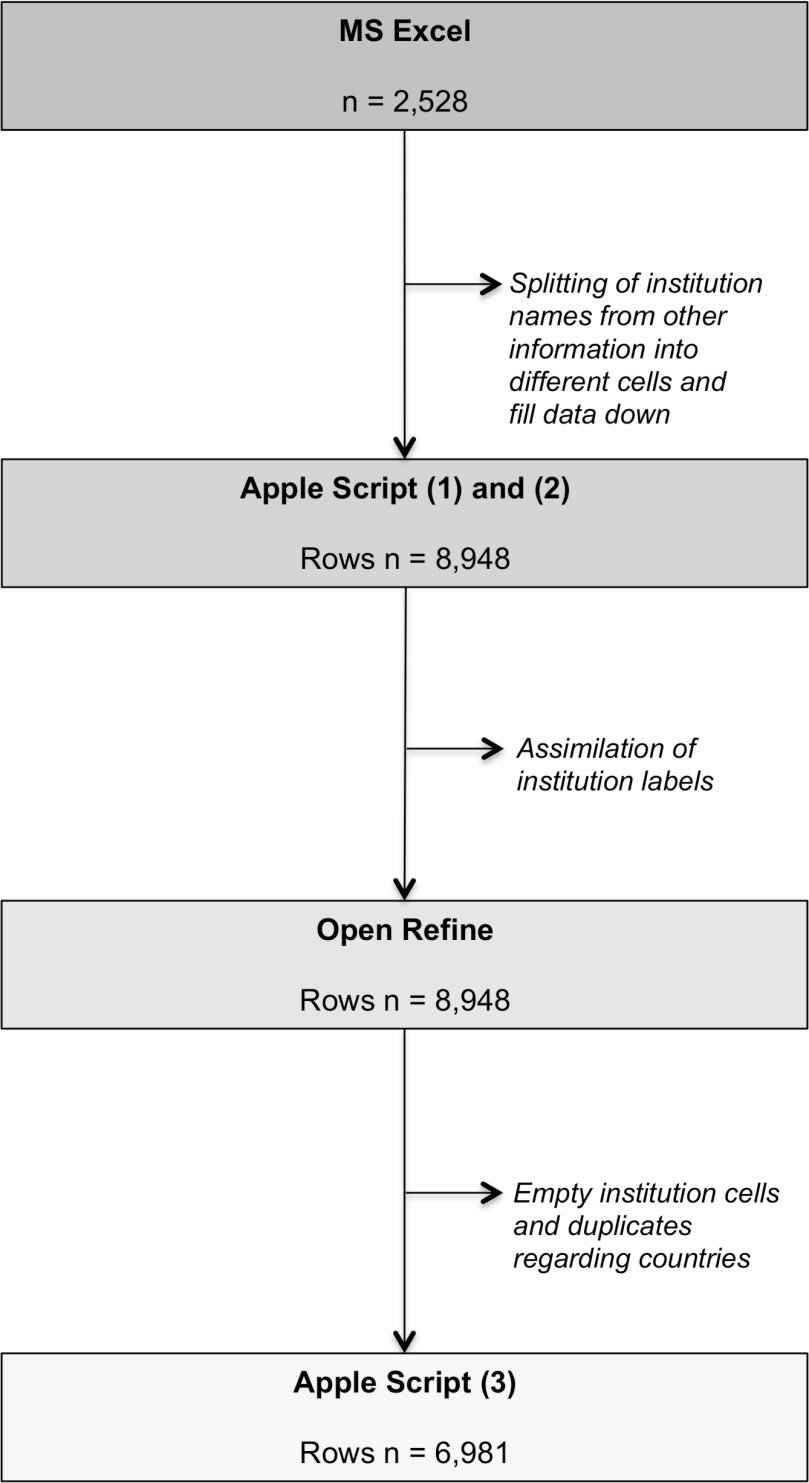
Flow chart, stepwise data processing using MS Excel, AppleScript and OpenRefine, by number of spreadsheet rows (one row per authors' affiliated institutions per publication).

Bibliometrics of 2,528 articles resulting for our WoS search were exported as tab-delimited data and imported into MS Excel as one bibliometric data set (Fig 2). In the raw data set each entry referred to one publication. We included data on title, authors, address of authors’ affiliated institution, publication year, source, language, document type, cited references, funding agency, publisher and subject category in further analysis. Other columns were deleted from the data set. Information on addresses of author’s affiliated institution, e.g. institution name, sub-departments and institution address including city and country, were split into separate columns.

### Data cleaning

Data processing and further cleaning was performed using the software AppleScript [22] and OpenRefine [23]. Name disambiguation, e.g. Centers for Disease Control and Prevention was abbreviated as CDC, Ctr Dis Contr and Centers Dis Cont, orders within names, e.g. Univ Washington and Washington Univ or name spellings, e.g., Univ Georgia, UNIV GEORGIA were identified and harmonised using OpenRefine algorithms or manually. Missing data, e.g. missing country information of an affiliated institution, were substituted by manual web search.

If an institution name appeared with addresses in different locations in the data set, e.g. WHO with location Switzerland and location Copenhagen e.g. due to different regional offices, different locations were considered for construction of the network to account for institutions international representations. Institutions duplicates originating from publications with multiple co-authors affiliated with the same institutions were eliminated to ensure a single weighting of institutions.

### Translating the data into a network

The free online application Table2net was used to extract network information from the refined data set to construct a Gephi readable file [24]. Network nodes (i.e. actors) are institutions named as authors’ affiliations in original research publications. Network edges are titles of joint publications from authors’ affiliated institutions.

### Measuring and visualisation instrument

The free software Gephi was used to calculate network metrics and visualise the networks [25].

Network analysis provides various tools and metrics in order to assess different notions of importance of individual nodes and node groups. As the simplest metric of centrality we calculated each node's ***degree***, as the sum of direct links to other nodes. Nodes with more direct connections are considered more central. The **average node degree** captures the number of actors that each actor is connected with on average. The **average weighted node degree** also takes the weight of a connection between a pair of nodes into account [26,27].

***Betweenness*** centrality measures the frequency with which a particular node lays on the shortest paths between all other node pairs. Therefore, nodes with a high betweenness are considered to have a broker position as they connect many other nodes and thus have a large influence on the transfer of items through the network, under the assumption that item transfer follows the shortest paths [26,28]. We used a betweenness calculation algorithm for weighted graphs as developed by Opsahl [29].

Besides positional properties of the nodes within the network, metrics are capturing topological aspects of the network as a whole. This information can provide an insight on the evolution at network level. ***Density*** measures were calculated to assess the connectivity of the network. The density of a network is defined as the total number of existing edges divided by the total number of possible connections. If edges exist between all nodes (density = 1) a network is considered completely dense [26,28]. Since density captures the probable feasible number of connections in a network, it is an indicator for possible community building [30] or innovation flow within a network [15].

Communities within the network were detected using Gephi’s ***modularity algorithm***. Modularity measures the degree of separation of a network into modules or clusters (communities). While a modularity value of 1 indicates that the actors separate perfectly into self-contained clusters, a value of -.5 suggest the opposite, a homogeneously connected network [27,31]. Networks with a high modularity score employ dense connections between nodes within the modules but sparse connections between nodes from different modules.

For visual presentation of network metric calculations we used Gephi's Force Atlas II algorithm in log-linear mode optimized towards hub dissuasion [32].

## Results

### Publications on EVD

Systematic search in WoS for publications containing “Ebola*” yielded a total of 4,587 publications between 1976 and 2015, including original articles (2,531), editorial material (659), news items (437), reviews (415), letters (325), meeting abstracts (157), corrections (36), notes (14), reprints (7), biographical items (4) and book reviews (2). Amongst the 2,531 original articles were 75 article proceedings and five article book chapters. Three of those publications appeared with anonymous authors and were therefore deleted for social network analysis (Figs 1 & 2).

**Fig 3:**
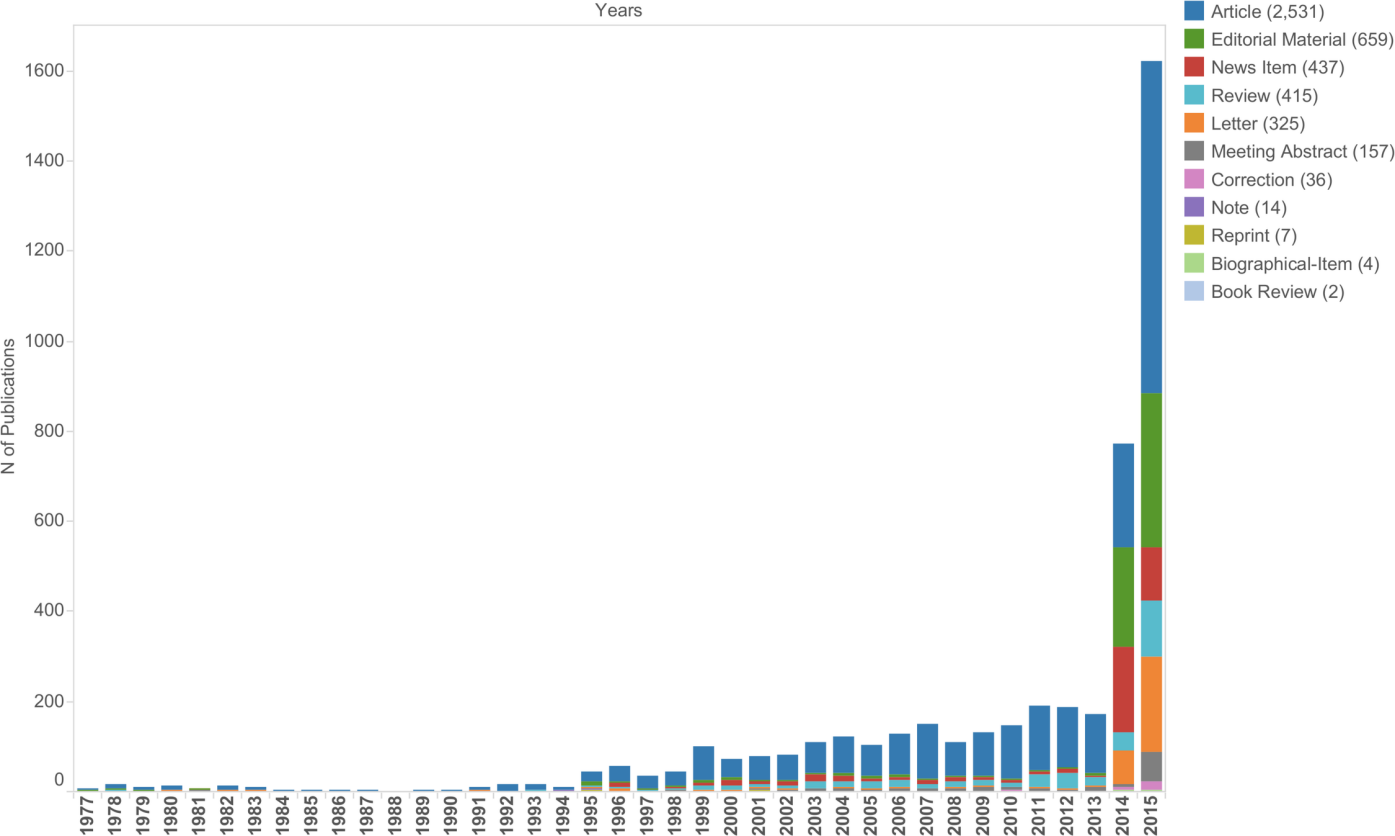
Articles of all authors from 1976–2015 (n = 4,587).

**Fig 4:**
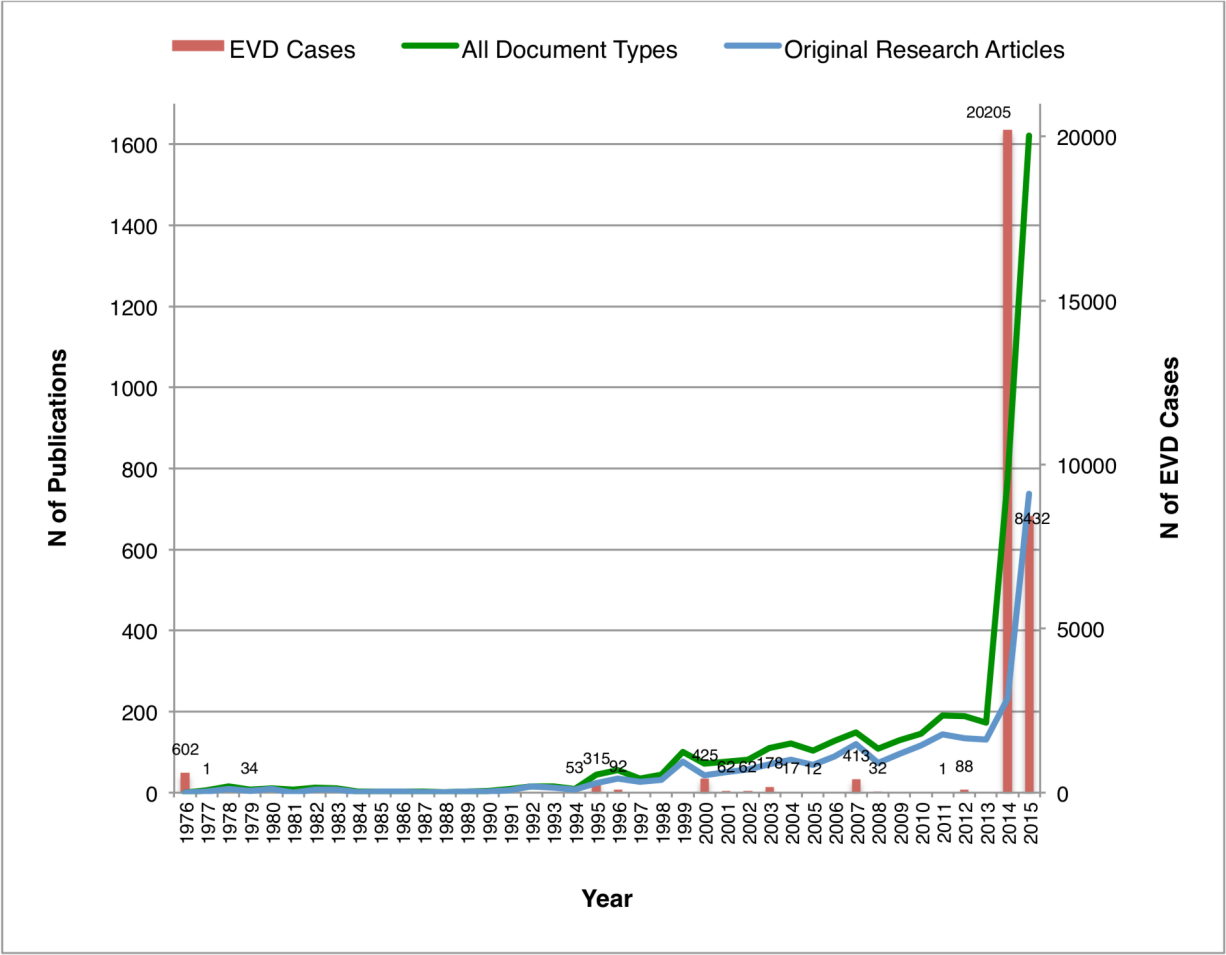
Annual EVD cases (n = 31,024), and number of publications on EVD from 1976 to 2015 [note the different ordinates on y-axes].

The first EVD research article was published in 1977, shortly after the first noted EVD outbreak in 1976. Only few EVD publications were visible until the early nineties, whereas from 1994 onwards the number of yearly EVD publications increased continuously (Fig 3). Since 1994 a higher frequency of EVD outbreaks were recorded and more EVD cases were being detected in almost every year. Several localised EVD outbreaks in Africa have occurred with up to several hundred cases. The initial EVD outbreak in 1976, with a relatively high number of reported cases (>600), was followed by only a small number of publications on EVD research. No EVD outbreaks were reported between 1979 and 1994 and hardly any publications were published on the topic. The number of publications increased gradually and continuously after the second outbreak in 1994, although compared to the 1976 outbreak only about one-tenth of cases were reported (Fig 4).

A substantial increase in EVD research publications occurred during the 2014/2015 West African outbreak. An almost 10-fold increase from 2013 (171), 2014 (772) to 2015 (1,621) was visible for almost all document types, but it was most pronounced for editorials (5, 220, 343), letters (1, 75, 213), news items (4, 190, 118) and meeting abstracts (9, 5, 66) respectively. An increase in reprints, notes, biographical items and book reviews was not detected.

### Global EVD research network

**Fig 5:**
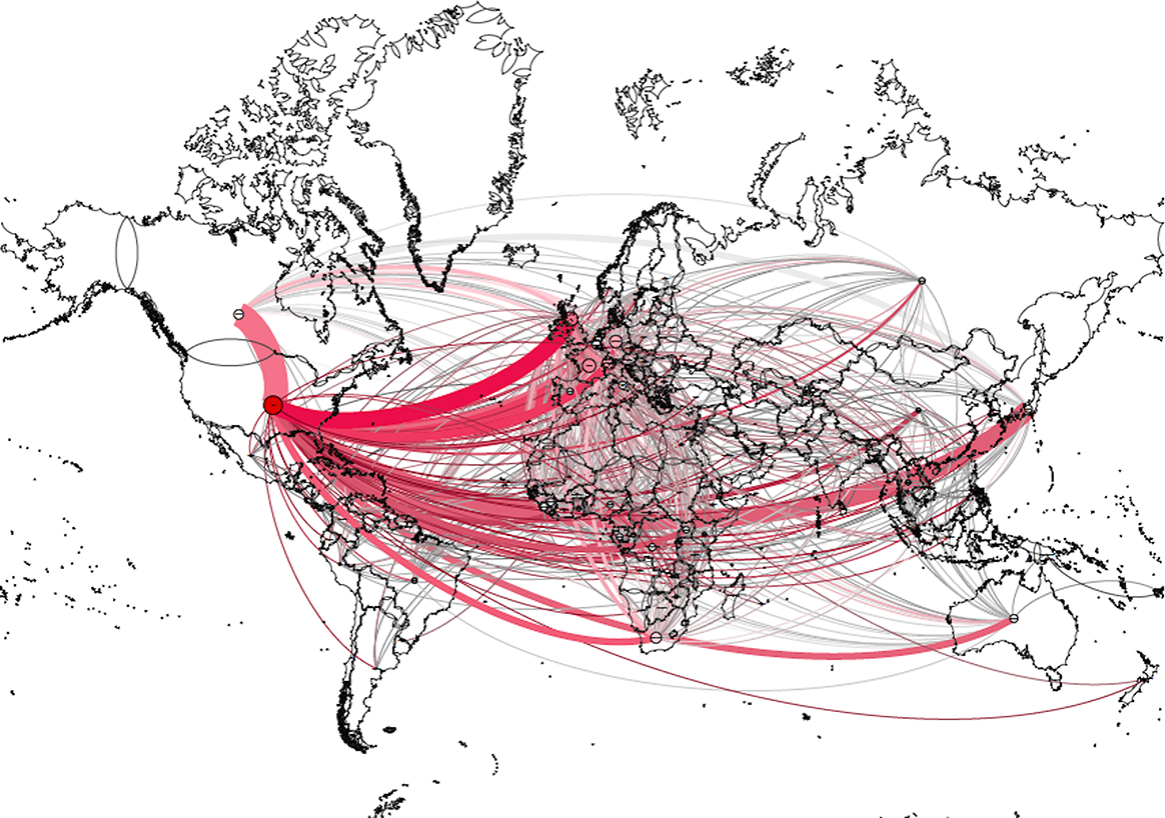
Global EVD research network, countries linked by cumulative co-authorships from 1976–2015, Layout: Geolayout.

**Fig 6:**
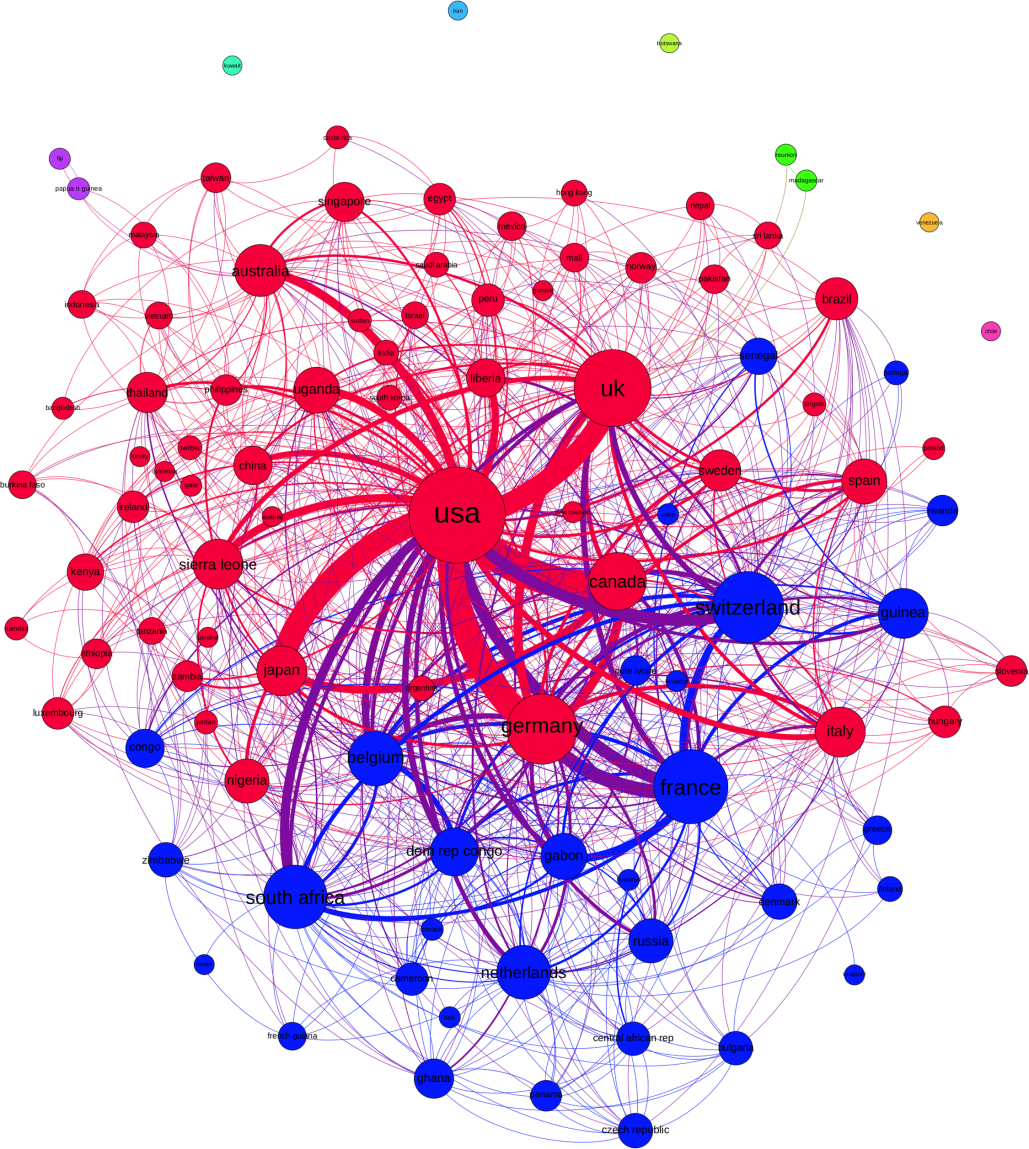
Global EVD research network, countries linked by cumulative co-authorship from 1976–2015, Layout: Force Atlas 2.

Bibliometrics of 2,528 original research articles were used for social network analysis. Based on their co-authors’ affiliated institutions a global network including institutions from 101 different countries with 704 connections was constructed (Figs 5 & 6).

Research institutions in the United States (US) are among the most highly connected institutions in EVD research (degree (d) = 80). They are mostly connected to institutions in Canada (d = 40) with an edge weight (ew) of 130 and Europe, especially Germany (d = 53, ew = 110), the United Kingdom (UK) (d = 60, ew = 90) and France (d = 57, ew = 51), but also to Japan (d = 32, ew = 99). Connections between US institutions and institutions in EVD affected African countries are less frequent (e.g. Guinea-USA ew = 14, Sierra Leone-USA ew = 32, Liberia-USA ew = 30). However, institutions in Sierra Leone and Guinea (both d = 32) and other African countries, especially Nigeria, Uganda and Ghana, are embedded in the global research network with connections to UK, Germany, France and Switzerland. The overall density of the global country-level EVD research network measures 0.15, with an average degree of 14.65 and an average weighted degree of 61.01.

Amongst all collaborations on country-level, nine research communities were identified using modularity-based community detection and visualised by different colours (Fig 6). The largest community (red) is centred around the US with strong collaborations to Canada, Germany and the UK, representing 59.41% of the co-authorships collaborations (weighted edges). Another large community is a (mostly francophone) European–African community (blue) representing 31.68% of all co-authorships connections.

### EVD research network on institution level

**Fig 7:**
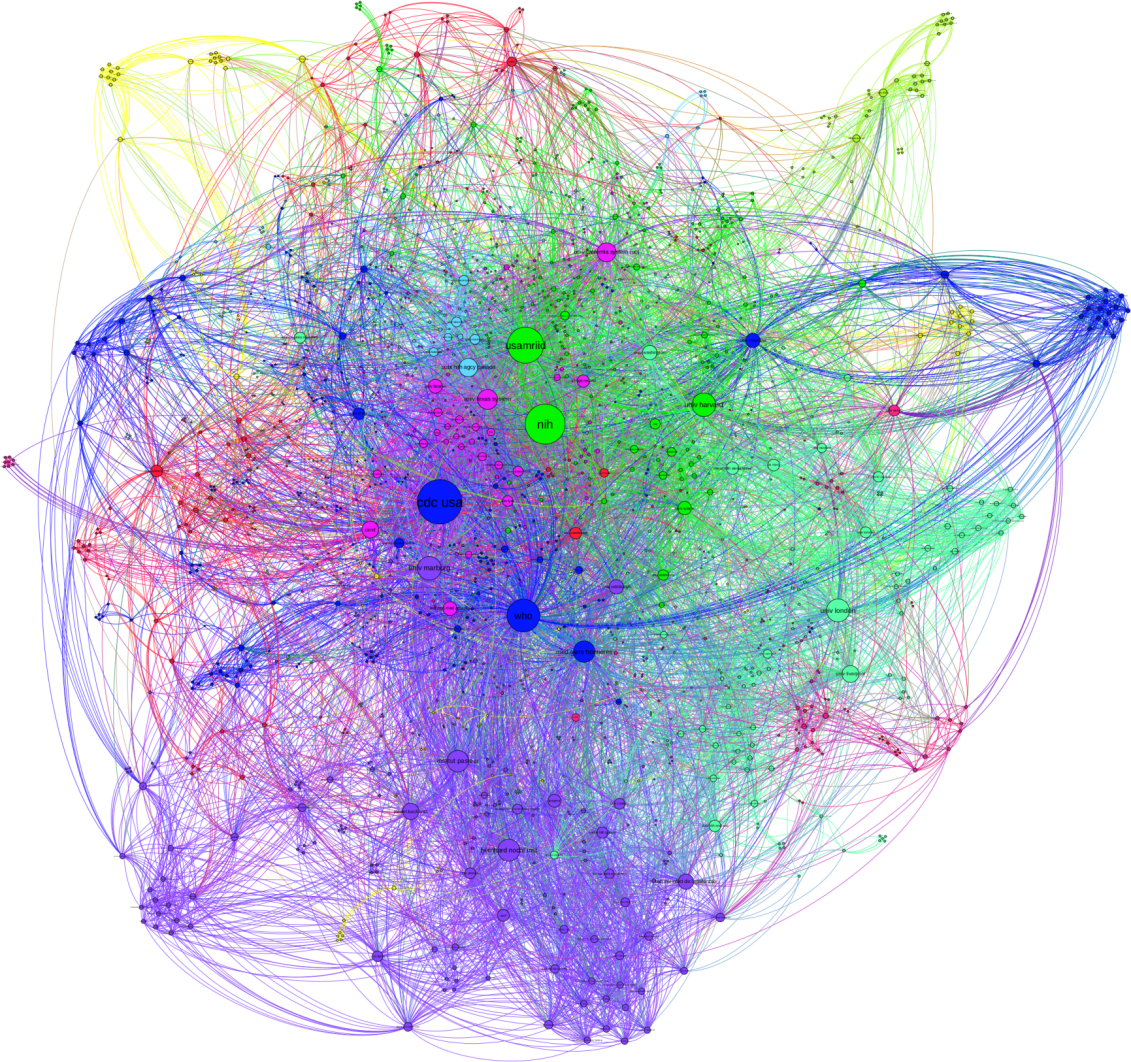
Cumulative EVD research network on institution level. Nodes sized by degree centrality. Research communities are colour-coded. Layout: Force Atlas 2.

Among all published original research articles between 1976 and 2015 a total of 1,644 co-author's affiliated institutions were named, which yielded 9,907 co-authorship connections in the overall research network (Fig 7). The main actors according to degree are the US government (CDC USA, d = 353; NIH, d = 315; USAMRIID, d = 283) and WHO (d = 256). Other prominent actors are from the US and European countries. Most central institutions are publicly funded (e.g. CDC USA, USAMRIID), government research institutions (e.g. BNI, ISERM), (mostly public) universities (e.g. Uni London, Univ Marburg) or international institutions (e.g. WHO) or non-governmental institutions (NGOs) (e.g. MSF).

Modularity analysis reveals 166 communities within the network (Fig 7), whereas the largest community (blue) represents 17.33% of the total network nodes and the second largest (green) represents 14.44% of the network nodes. Numerous smaller and less connected communities exist in the periphery, with some being entirely disconnected from the main network.

### Network development over time

**Fig 8:**
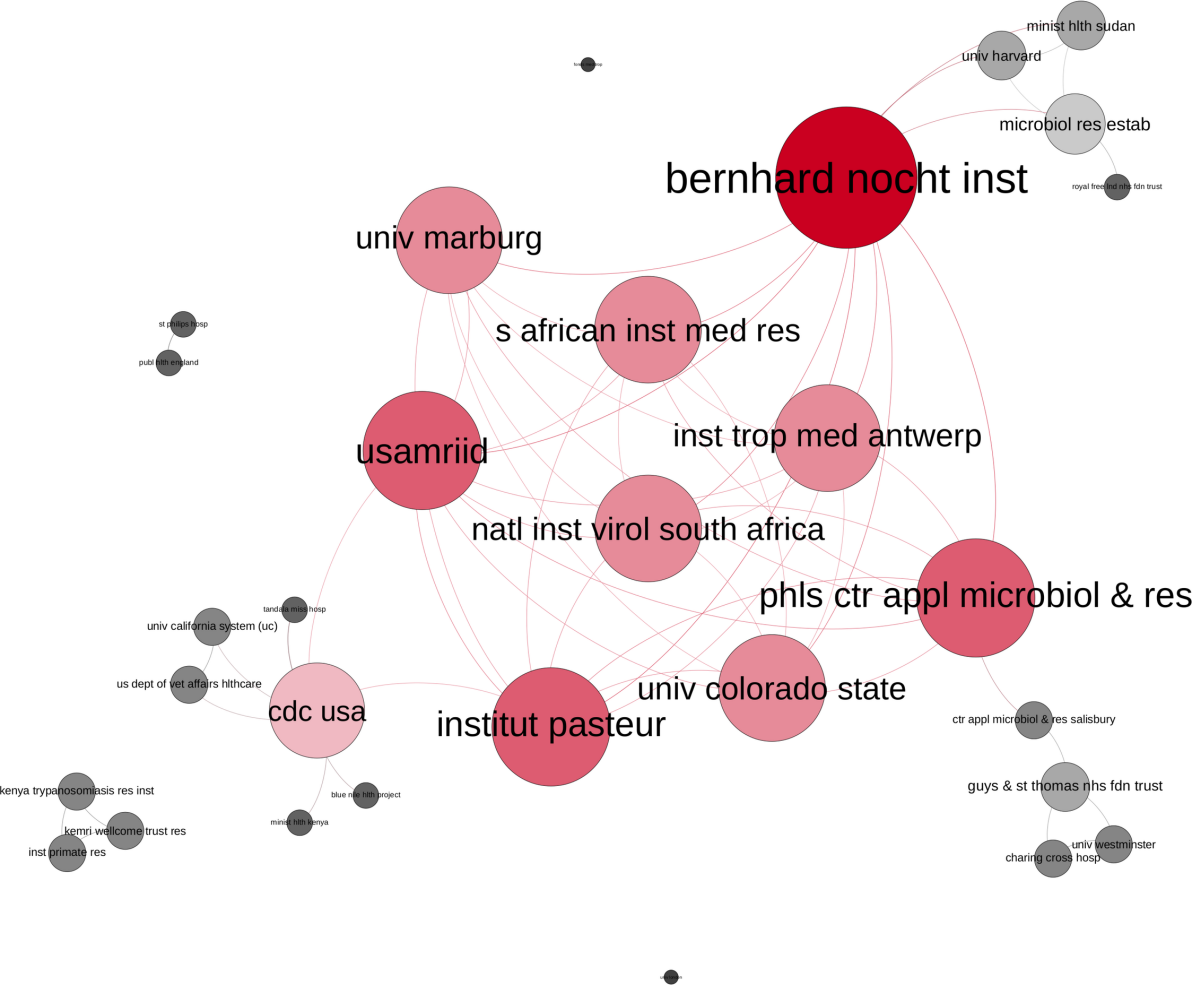
EVD research network. Publications from 1976–1985 by degree centrality (colour and size of nodes), Layout: Force Atlas 2.

**Fig 9:**
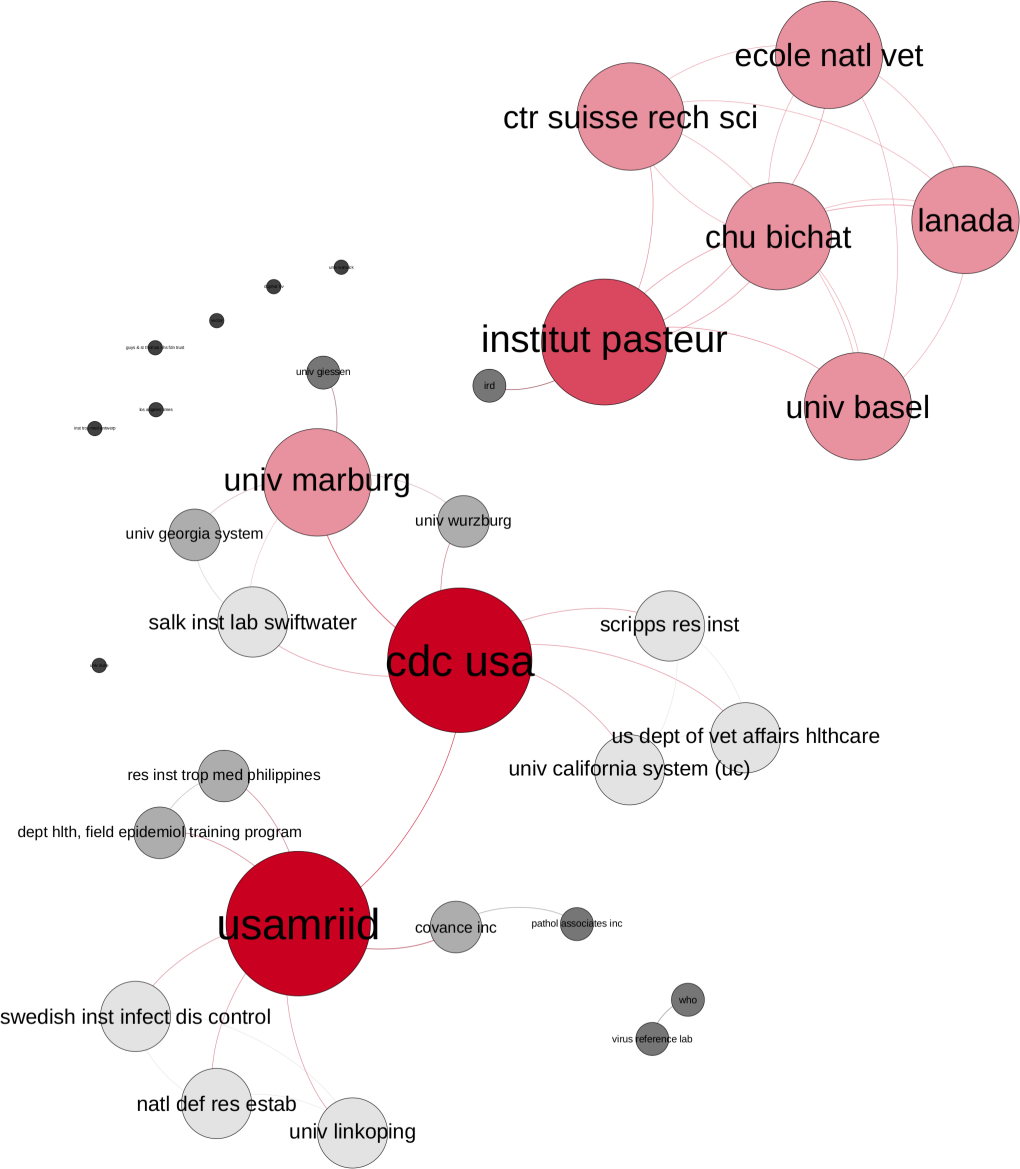
EVD research network. Publications from 1986–1995 by degree centrality (colour and size of nodes), Layout: Force Atlas 2.

**Fig 10:**
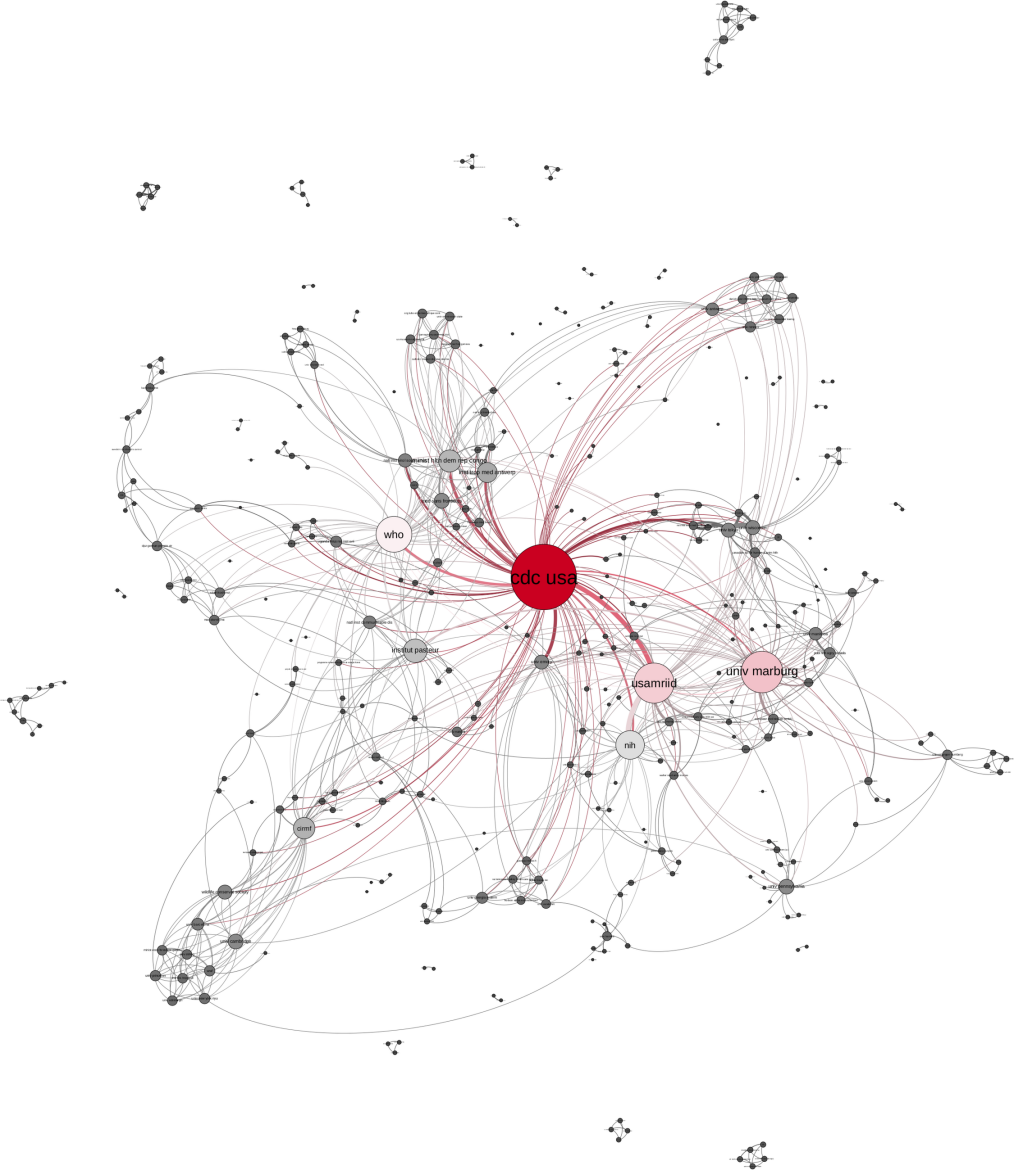
EVD research network. Publications from 1996–2005 by degree centrality (colour and size of nodes), Layout Force: Atlas 2.

**Fig 11:**
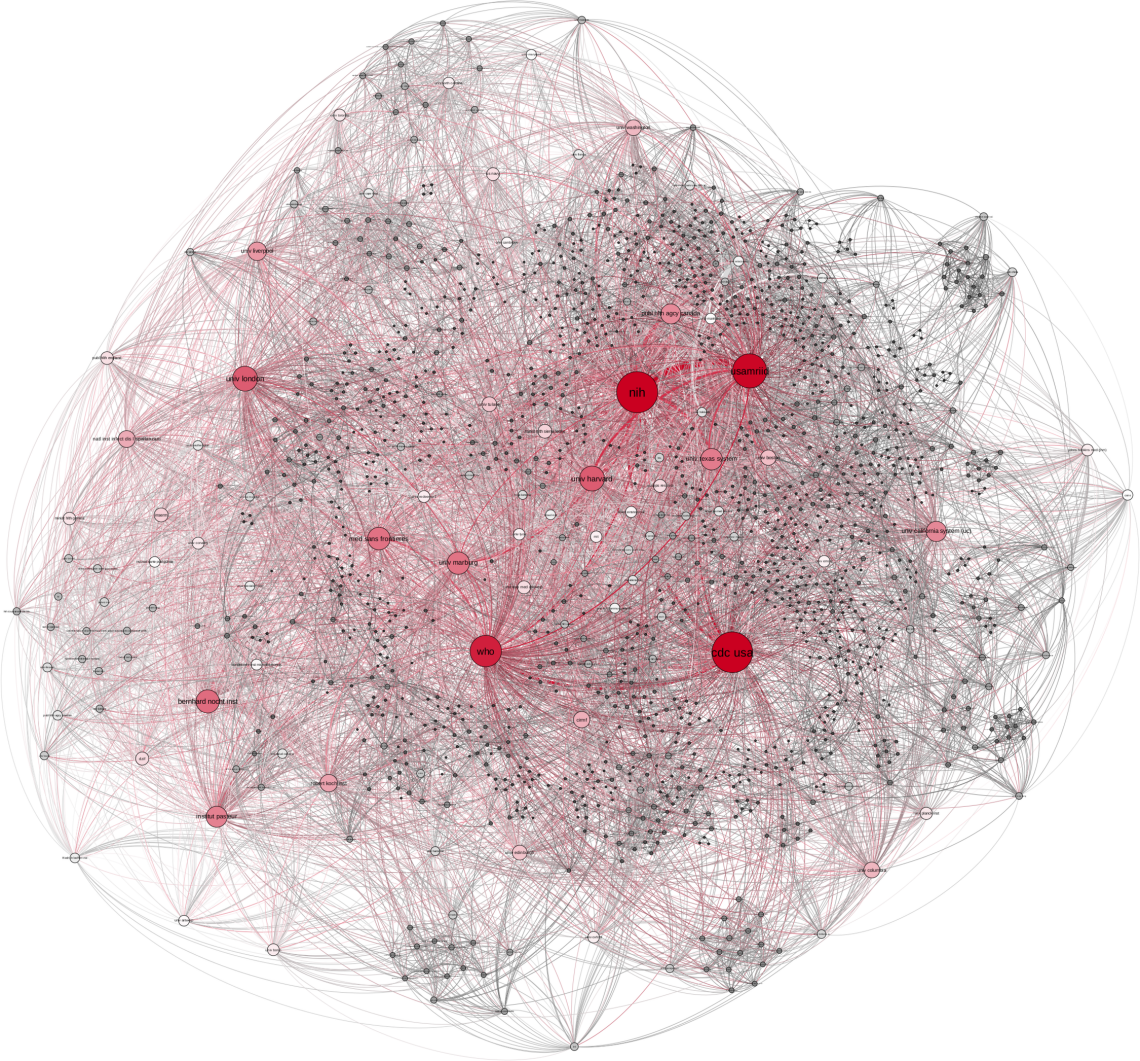
EVD research network. Publications from 2006–2015 by degree centrality (colour and size of nodes), Layout Force: Atlas 2.

The temporal development of the research network is visualised over four 10-year time periods (Figs 8, 9, 10 & 11).

In the first decade 1976–1985, (Fig 8) the network consists of only a few actors, with one large central cluster surrounded by four smaller clusters. The German Bernhard-Nocht Institute (BNI) has the highest centrality degree (d = 11), closely followed by the Institut Pasteur, PHLS Center for Microbiology and Research (Salisbury, UK) and USAMRIID. The CDC USA is a central institution (d = 7) of a smaller cluster, publishing with African partners (Kenyan Ministry of Health) others. Smaller research groups in Kenya (Kemri Wellcome Trust, Institute of Primate Research, Kenya Trypanosomiasis Research Institute), UK and US published together, but had no connections with others.

In the second decade 1986–1995, (Fig 9) two larger, but separate, research communities evolved. One francophone French-Swiss-African community with a homogenous structure in which the Institut Pasteur published mainly with the University of Basel, Institut de recherche pour le développement (IRD), Ecole national veterinaire Lyon and the Hospital Bichat Claude Bernard Paris. The other community consists mostly of American and German institutions, with three main actors (USAMRIID, CDC USA and the University of Marburg), where the USAMRIID and CDC USA connect this community. During this period the WHO had its first appearance as a disconnected actor. All institutions in the network of the second decade are public entities.

With the occurrence of new EVD outbreaks in 1994/1995 the EVD research network grew in the third decade 1996–2005, (Fig 10) into a star-like structure with surrounding chains. During this decade the CDC USA evolved as the most central actor (d = 87). The University of Marburg (d = 54), USAMRIID (d = 52), WHO (d = 46) and NIH (d = 36) remain central but less prominent actors.

The network of the fourth decade 2006–2015, (Fig 11) is skewed by publications in 2014/2015. During this time only few public research institutions and university actors dominate the research collaborations but numerous new actors appeared. Prominent cooperation exist between CDC USA and WHO and CDC, NIH and USAMRIID. While the transnational WHO was well embedded in the network over these last two decades, all main network actors are public institutions, mostly from the US and European countries.

### Network metrics over time

While the global EVD research network remains relatively consistent in the first two decades, the third and in particular the forth decade shows substantial overall increase in the number of institutions and the links between them (Table 1). Simultaneously the average node degree and weighted node degree increased over time, which indicates a growing number of collaborations and research activity per institution.

**Table 1:** SNA parameters and metrics of the global EVD research network, for overall network and stratified by 10-year periods.

	Overall	10-year periods			
	(1976–2015)	(1976–1985)	(1986–1995)	(1996–2005)	(2006–2015)
**Publications**	2528	45	74	536	1873
**Nodes** *	1,644	30	33	357	1,489
Edges**	9,907	60	43	882	9,176
**Node degree (avg.)**	12.05	4	2.61	4.94	12.31
**Node degree (weighted avg.)**	17.89	4.07	2.85	6.89	17.95
**Network diameter**	8	6	5	7	8
Path length (avg.)	3.02	2.74	2.31	2.99	2.99
Shortest paths	2,032,226	514	316	60,530	1,634,748
**Density** ***	0.007	0.138	0.081	0.014	0.008
**Modularity** ****	0.46	0.46	0.65	0.61	0.44
Communities	166	8	11	69	154

*Authors’ affiliated institutions

**Co-authorship of authors’ affiliated institutions by joint publication

***1 means completely dense

****with resolution 1.*0*

The decreasing density of the network over all decades indicates a decreasing number of realised edges between nodes relative to the total number of possible edges. The increasing average node degree implies a growing number of research connections per institution. The number of communities increased in line with number of nodes. The high modularity values show that the solutions of the community detection algorithm reflect the substructures of the graph well, i.e. the increase in communities is unlikely to represent a sheer increase in volume, but rather seems to capture an evolution of the field of EVD into several smaller communities.

### Degree centrality distribution

**Fig 12:**
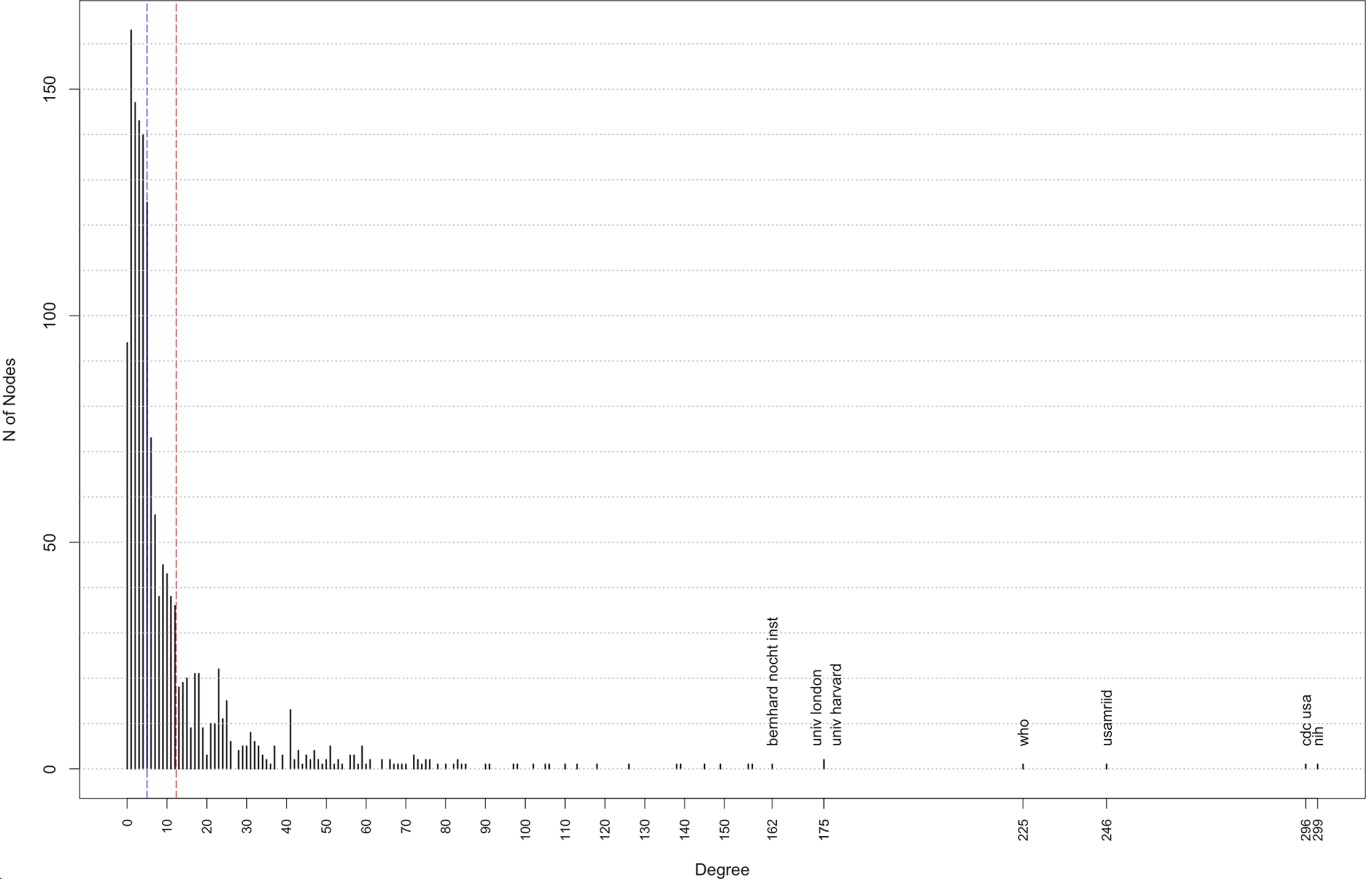
Degree centrality distribution, network from 2006–2015 [median (blue line), mean (red line)].

A degree distribution analysis of the EVD research network in the fourth decade shows a skewed node-degree distribution (Fig 12). While almost 100 nodes appear with a degree of zero (d = 0), indicating no collaboration at all, only few institutions have a very high degree above 160 (mean 12.24; median 5). Most institutions had a degree of less than five (d≤5) as they were named as affiliations by authors of few publications by authors that published with only few co-authors. The few very well connected institutions, such as NIH and CDC USA, are the key actors in this period. In fact the CDC USA has maintained a very central position in the network over all time periods. The private NGO Médecins Sans Frontières (MSF) has only recently emerged within the network and is centrally embedded with a high degree (d = 157).

#### Main actors in the global EVD research network

Calculating degree and betweenness centrality for all network nodes allowed ranking and identifying the most central network actors (Table 2). Exclusively publicly funded institutions are among the top 10 ranked institutions (degree and betweenness centrality), while US research institutions are central institutions in the network. The CDC USA is the institution with most collaborations (highest degree) and linking most institutions (highest betweenness centrality), closely followed by USAMRIID, the NIH and the World Health Organization (WHO).

**Table 2:** Top 10 ranking of institutions by degree and betweenness centrality.

Ranking	Top 10 institutions (by degree centrality)	Top 10 institutions (by betweenness centrality)
**1.**	CDC USA (353)	CDC USA (173,132.04)
**2.**	NIH (315)	NIH (130,496.07)
**3.**	USAMRIID (283)	USAMRIID (121,169.95)
**4.**	WHO (256)	WHO (82,398.38)
**5.**	Univ Marburg (182)	Univ Marburg (47,811.38)
**6.**	Univ Harvard (181)	Univ Harvard (43,055.20)
**7.**	Univ London (176)	Univ California System (41,661.66)
**8.**	Bernhard Nocht Inst (168)	Bernhard Nocht Inst (39,446.48)
**9.**	Institute Pasteur (164)	Univ London (38,083.88)
**10.**	Médecins Sans Frontières (164)	Univ Texas System (36,863.03)

No institution from an African country ranks for degree or betweenness centrality amongst the top 10 institutions (based in the US and Europe), whereas MSF ranks for degree among the top 10 entities.

## Discussion

### Description of the network

Since the first reported EVD outbreak in 1976 until today the total number of publications on EVD in WoS has exceeded more than 4500 publications, of which 2528 were original research articles. Like in scientometric analyses we used joint publishing as a proxy indicator of scientific collaboration [17] and thus knowledge exchange for our SNA of the co-authorship network [11,13,30]. Indeed for the EVD overall network we identified research contributions from 1,644 research institutions in 101 countries; most actors are indeed coming from the US [17]. Since 1994 EVD research publications have increased continuously, steadily and independently of the major West African outbreak. This growth in publications is mirrored by a growth in the number of institutions (from 30 to 1,489) and edges (from 60 to 9,176) and therefore on-going network growth accompanied by a decreasing network density. The overall network is an extensive aggregation of 166 different communities with a clearly dominant anglophone and francophone community. This same dominance is seen when analysing the most central actors by degree and betweenness centrality both confirming the dominance of 10 institutions in powerful, control or broker positions in the network [11,33,34].

### Analysis of the network

The pattern of a growing EVD network in size but with a reducing density is characterised by some outliers (106 institutions not connected), frequently less connected contributions from developing countries and the private sector, but with a strong and stable core of dominant or ‘central’ institutions. These characteristics of the network are supported by many of the analyses we performed. For example the relatively and increasingly poorly connected nature of the network (network density), the heavily skewed node degree distribution with the median node degree remaining rather constant, the relatively compact nature of the network (path lengths) and the strong centralisation showing a dominance of a few very strongly connected actors and many poorly connected actors.

Although we acknowledge that our analysis is weakened by the absence of a comparator network (a common challenge in emerging research fields), we also believe that our analysis bring some added value. For example SNA metrics for the overall network shows a density of 0.007 and calculating network density for each decade individually showed progressively decreasing density from 0.138 in the first decade to 0.008 in the last decade. While this is largely influenced by both the size (the more actors a network includes, the more difficult it is for all actors to be connected) and also the correspondingly rapid growth in the network (connections take time to build), we still believe that these figures should raise questions about whether the network–and therefore research outputs–could benefit from greater connectivity and linkages and in doing so greater optimise knowledge transfer and the spread of innovation [15]. The node degree distribution (for the last decade from 2006–2015) further confirms both the observed increase in the average node degree is attributable to only a few central actors whereas the overall network was not well connected in this period. Thus, the network growth during the 2014/2015 epidemic diluted connectivity, at a time when collaboration was arguably most needed.

These observations are built on when we look further at the node degree distribution for 2006–2015. This confirms that while most actors only had few connections during this time, some actors are extremely connected. This distribution form has been described as “power law” or “scale free distribution” and is typically observed amongst poorly connected networks [35,36]. This ‘concentrated core’ is corroborated by the high number of the average weighted node degree (17.89), in contrast to the average node degree (12.05), which is also an indicator that some actors in the EVD network are connected more strongly to each other than others due to repeated publishing [27]. It shows that these actors have on-going collaborations, share research results intensely by jointly publishing—but focus sharing amongst their co-authors. This latter finding is something confirmed by our SNA results, which show strong centralisation amongst six institutions (CDC USA, NIH, USAMRIID, WHO, the University of Marburg and the University of Harvard), suggesting that knowledge is mostly exchanged within the network between and/or through these actors. Centrality is a measure of power in SNA [37], this is especially the case for our central actors whose knowledge broker status is confirmed with regard to EVD research due to their high degree and betweenness centralities.

Additionally, observation of the path lengths reveal further insight into the efficiency of information exchange, with the shorter the average path length of a network diameter, the more efficient is information exchanged within the network structure [26,35]. We found that the average paths lengths (3.02) of the overall network is lower than the average node degree (12.05), indicating both that some institutions have a lot of direct neighbours and that on average nodes can reach other nodes by crossing only two other nodes. The network diameter (8.0) suggests that sub-graphs within the network do not span more than across a chain of eight nodes. Taking both aspects into account this implies that the overall structure of the network is characterized by isolated and weakly connected components, i.e. localized small networks that have only few relations amongst each other.

### Network actors

Although our study cannot, unfortunately, reveal anything about the ‘type’ of research conducted, observations on the type of research institution maybe serve as a proxy for this insight. Two notable observations here were both the relative underrepresentation and disconnectedness in the overall network of both research institutions from affected countries and the private sector. Among the unconnected nodes appear some private industry actors (e.g., Novartis Vaccines, Biohelix Corp, Baxter Bioscience and Oravax Inc.), in addition to African universities such as the University of Benin and the University of Mbarara. While there may be many good reasons that explain the disconnectedness, for example proprietary restrictions to collaboration (in the case of industry), new entrants to the field or for resource-related barriers to International collaboration. This observation remains significant for a number of reasons, presumably both of these actor types posses’ unique and distinct knowledge and capabilities that could diversify and strengthen the expertise within the network if better and more broadly integrated, this is likely even more the case during a public health emergency of international concern. Also, this ability to identify disconnected but valuable nodes, demonstrates a great added value of tools such as SNA. Finally the recent entry into the network of non-traditional research actors such as MSF should be welcomed, especially as endemic country capacity is being developed and integrated into international networks, due to their unique position as being close to patients in the field yet able to advocate–distant funders–on the need for a well-supported, needs-driven research agenda [5,38].

### Network–implications for policy

We believe the structure, nature and evolution of the international EVD research network described in this paper presents some learnings for policy. Looking positively, the network itself has maintained a similar structure–a relatively compact network with a few consistent actors at its core–over the four decades studied, implying it is a stable constellation. This institutional memory provides a solid foundation for knowledge maintenance over time, indeed without central actors networks might be disrupted and knowledge exchange hampered [30]. The growth in the network over time through the entry of new actors, particularly since 2014/2015, is positive as it likely indicates the arrival of new ideas and approaches. However although collaboration has increased over time, our analysis found that the network remains relatively poorly connected. Hence there may be an additional role for the ‘central actors’ to expand their role beyond a hub for dissemination and exchange into a facilitator for integrating the newer actors and expertise into the network. Additional opportunities presented by the network analysis include: a reflection on the, perhaps, over-reliance or vulnerability to the network of all of the ‘central actors’ being public government or university institutions. The importance of predictable, sustainable, funding flows to their continued role as network ‘brokers’ feels more exposed in these current financially and politically turbulent times. While the dominance of these institutions is not surprising, we assume that they have the infrastructure, capability and public-financing, it may represent a weakness in two respects: firstly, with respect to its insufficiently diverse expertise mix, particularly with respect to the translation of this research into the development of tangible, context-relevant tools and capacity building in affected countries [8,39]; secondly, with respect to the risk of over-centralising expertise, resulting in the stifling or suppression of innovation and growth and development of new ideas.

Finally, in small research areas for diseases predominantly impacting the lives of those in low-income countries such as EVD, the inherent market failures indicate that this reliance of public-financing will likely continue [Wölfel in: 3,5–7]. Given this, we believe, that a valuable insight from our study is to observe ways in which the network efficiency could be enhanced to extract greater patient-impact from the public financing inputs. For example: focused efforts on integrating new collaborators into the network, provision of tools to enhance the productivity and improved transparency and sharing of research data [9,40] the identification of expertise gaps and targeted filling of these gaps and lastly, but perhaps most importantly, National alignment, focus and financing coordination (strategic research planning) around the globally agreed prioritised research agenda [41]. Although many of these calls have already been made by many actors, particularly since the 2014/2015 EVD outbreak we believe this study represents an important empirical tool to support these calls and inform National and global policy development as the global community works to avert the next EVD outbreak.

### Limitations

The use of bibliometric data has intrinsic limitations and restrictions related to any analysis of secondary data and where data ceases to provide information, in particular in relation to content or results of published research.

Two major limitations to our study were identified and previously highlighted. The first being the absence of other publications with which to contextualise and compare our results. This absence of relativity in our conclusions limits the comparative value of our findings although the absolute data remain valid. Although SNA is increasingly being used as a tool to analyses research areas it remains a relatively new field so we are optimistic that this is a time-limited constraint.

Secondly, we acknowledge that our study would be greatly enriched by an ability to analyse the data by ‘type’ of research not only type of publication i.e. basic, applied, clinical, implementation research, translation, health systems etc. However, at present, this is not a search field within WoS, so we were unable to attain the source data. Should key, public, medical, search engines enable this in the future, SNA such as ours would be an even more powerful tool to provide insight into research focus and productivity. This analysis we believe would have great value–supplementing existing financing and development pipeline analyses [42,43]—in providing a more granular understanding of product development gaps and the persistent absence of tools for the prevention, diagnosis and treatment of EVD [6,44]. Our analysis of decreasing network density over time could have been further triangulated with the use of an additional metric such as the percentage of the giant component or the clustering coefficient. Other limitations include reporting delays and the possibility that some publications were not included in the WoS database, however sample testing of other databases, including PubMed.gov, did not reveal other publications on EVD.

Although the impact of missing publications was likely small future studies could aggregate studies from diverse databases and in particular try to assess contribution of private industries R&D. Despite manual and automated attempts to resolve challenges with institution name cleaning and disambiguation it cannot be excluded that some actors and/or relationships were not captured or were captured incorrectly. Although unlikely, errors of the software used cannot be completely excluded and different algorithms might lead to different presentations of results. Therefore network visualisations should be critically assessed in context to minimise misinterpretations. We further note that GeoLayout visualisation can be misleading since it locates the African continent in the map centre and visualised edges may overlap nodes. For this reason a country distribution was processed additionally with Force Atlas 2. The use of only free available software and easy accessible bibliometric data from WoS both facilitate the easy reproducibility of our study.

### Conclusions

We conducted the first systematic landscaping of published EVD global research bibliometrics using SNA tools for analysis and visualisation.

Since 1976 Ebola outbreak EVD research, numbers of authors and affiliated institutions and links between them are constantly increasing, mostly independent from outbreaks and in-particular in the past two decades.

The overall EVD research network is organised around a few co-authoring key actors, mostly publicly financed. Low network density indicates room for increased cooperation between institutions, in-particular links to less connected and more peripheral institutions could foster knowledge exchange and innovation. Key network actors, such as the CDC USA, maintained network coherence over time–and probably kept EVD research on-going. Limited scientific collaboration of research organisations from LMIC and the private industry, and how they utilise their expertise and knowledge, is neglected.

However, the absence of effective treatments for EVD questions the existing EVD research network efficacy and efficiency and suggests the need for both direction and structure to optimize the network to focus on research relevant for treatments. Since most institutions in the global network are publicly funded, guidance to direct and re-orientate research might be facilitated by funders (through calls targeting knowledge and translation gaps) and be offered by supranational policy setting entities such as WHO and its Global Observatory on Health Research and Development.

Further in-depth quantitative and qualitative analysis, e.g. text mining of publications abstracts, analysis of EVD research study methods and separate R&D product pipeline analysis, is recommended to ensure empirically based strategic research guidance and relevant to EVD product development.

In any case, SNA of co-authorship networks is an innovative tool to evaluate research collaborations between individuals, organizations and countries, contributes to the understanding of the evolution of research networks and should be used for strategic research planning and a regular monitoring.
